# The complete mitochondrial genome of leafhopper *Koreocerus koreanus* (Matsumura, 1915) (Hemiptera: Cicadellidae)

**DOI:** 10.1080/23802359.2021.1962765

**Published:** 2021-08-09

**Authors:** Kai Yu, Hu Li

**Affiliations:** Shaanxi Key Laboratory of Bio-resources, School of Biological Science & Engineering, Shaanxi University of Technology, Hanzhong, China

**Keywords:** Cicadellidae, *Koreocerus koreanus*, mitogenome, phylogeny

## Abstract

In this study, the complete mitochondrial genome (mitogenome) of *Koreocerus koreanus* (Mastsumura, 1915) was sequenced with 16,428 bp in length, which consisted of 37 genes including 22 transfer RNAs genes (tRNAs), 13 protein coding genes (PCGs) and 2 ribosomal RNAs genes (rRNAs). Except *COX1* and *ATP6* initiated with the TTG, other PCGs started with ATN. The *COX1*, *ND4* and *ND4L* terminated with an incomplete T, while the remaining PCGs stopped with TAA. Excluding *tRNA-Ser1* lacking a DHU arm, 21 tRNA genes with secondary structures were typical of cloverleaf structures. Phylogenetic analysis based on the complete mitochondrial sequences of 28 species (26 Cicadellidae and 2 outgroups) showed that *Koreocerus koreanus* was clustered into the subfamily Idiocerinae, the complete mitogenome of *Koreocerus koreanus* provides information for further study of Cicadellidae evolution.

The leafhopper species *Koreocerus koreanus* (Mastsumura, 1915) belongs to the subfamily Idiocerinae (Hemiptera: Cicadellidae) (Zhang 1990), it is a common species in Cicadellidae distributed in the Palearctic and Oriental regions. According to our observations, *Koreocerus koreanus* usually appears to feed on the willow. The body length of the species is 5.0–6.3 mm, and it has pale green pronotum, and yellowish-green scutellum, its male genitalia connective is Y-shaped, and the aedeagus is back curved (Zhang et al. 2019).

In this study, the complete mitochondrial genome of *Koreocerus koreanus* was first sequenced and described. Combined with the existed relative mitogenomes of Cicadellidae in GenBank, the phylogeny of Idiocerinae and Cicadellidae were primary analyzed.

The specimen of *Koreocerus koreanus* in this study was collected from Huayang National Nature Reserve, Shaanxi Province, China (107°58′23″E, 33°35′48″N), and then stored in absolute ethanol at −20 °C and deposited in the Museum of Zoology and Botany, Shaanxi University of Technology, Hanzhong, China (SUHC) (Juan Li, 13379130339@163.com; Yan Yan, 18799512013@163.com) the accession number is 202001y4.

Genomic DNA was extracted by a TIANamp Genomic DNA kit (Tiangen, Beijing, China) and the extraction steps were strictly in accordance with the instructions of DNA extraction kit for gene tissue. The complete mitochondrial genome was sequenced using the Illumina NovaSeq 6000 platform, and assembled by Geneious Prime (Kearse et al. 2012).

tRNA genes were predicted by ARWEN1.2 (Laslett and Canback 2008), rRNA genes were determined by the boundary of tRNAs and aligned with homologous genes from related species in the GenBank, control region was located between *12S* rRNA and *tRNA-Ile*. PCGs were annotated by aligned with homologous genes from Cicadellidae species.

The complete mitogenome of *Koreocerus koreanus* was a circular molecule containing 16,428 bp in total length, which was deposited in GenBank (accession number is MZ169558). All 37 mitochondrial genes were typical and presented in the sequence including 13 PCGs, 22 tRNAs, 2 rRNAs and one control region, of which, 14 genes were located in the N-strand including 4 PCGs (*ND1*, *ND4*, *ND4L*, and *ND5*), 8 tRNA (*tRNA-Gln*, *tRNA-Cys*, *tRNA-Tyr*, *tRNA-Phe*, *tRNA-Pro*, *tRNA-His*, *tRNA-Leu2*, and *tRNA-Val*), and 2 rRNA (*16S* and *12S*), 23 genes were encoded in J-strand. No gene arrangement was found in this sequence, the characteristic of *Koreocerus koreanus* mitogenome was similar to that of *Drosophila yakuba* (Clary and Wolstenholme 1985). 6 intergenic spaces and 18 gene overlaps were identified, the length were varying from 1 to 5 bp, and from 1 to 6 bp, respectively.

The nucleotide composition of *Koreocerus koreanus* was significantly biased toward A and T (A 44.9%; T 30.6%; C, 14.7%; G of 9.8%), and the AT content was 75.5%. With the exception of *COX1* and *ATP6* which initiated with the TTG, other PCGs started with *ATN*. The *ND4* and *ND4L* terminated with an incomplete T, while the remaining PCGs stopped with TAA. The tRNA genes ranged from 62 bp (*tRNA-Ala*) to 74 bp (*tRNA-Leu1*). Except *tRNA-Ser1* lacked DHU arm, 21 tRNA genes with secondary structures were typical of cloverleaf structures.

A phylogenetic tree based on the available mitogenome sequences of 26 Cicadellidae species as ingroups and 2 species of Membracidae downloaded from GenBank as outgroups was generated using maximum likelihood method (ML) under Kimura 2-parameter model with 1000 bootstrap replicates by MEGA6 software (Tamura et al. 2013). *Koreocerus koreanus* was clustered into the subfamily Idiocerinae (Figure 1). This topology supports that Idiocerinae is a monophyletic group, which is consistent with previous studies (Wang et al. 2018; Di et al. 2020). The complete mitogenome of *Koreocerus koreanus* will promote future phylogenetic study of Cicadellidae.

**Figure 1.**
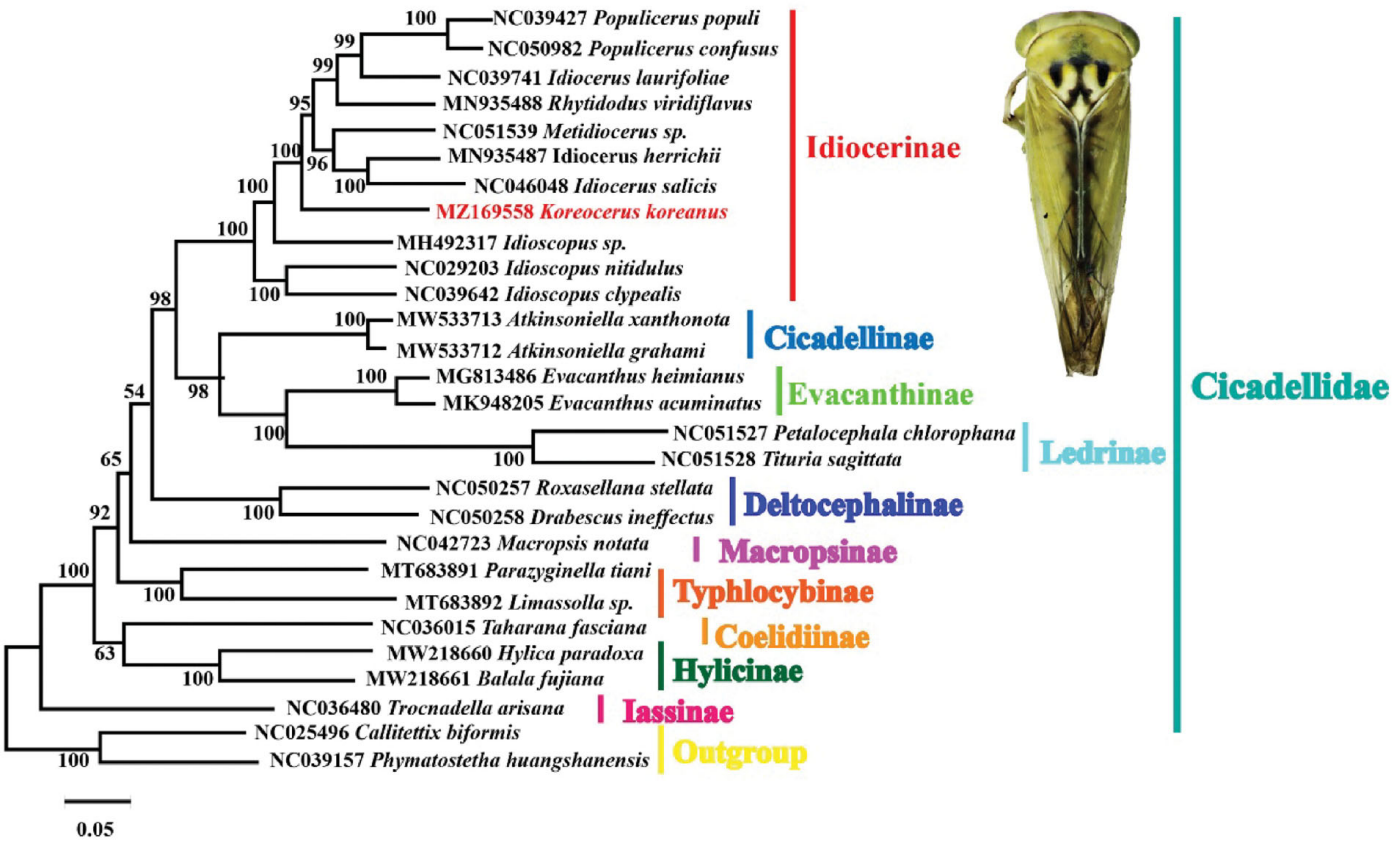
Phylogenetic tree of Cicadellidae based on 28 complete mitogenomes using Maxumum likelihood (ML). Numbers at the nodes represent bootstrap values. The adult imaging of *Koreocerus koreanus* is shown in the plate.

## Data Availability

Mitogenome data supporting this study are openly available in GenBank at nucleotide database, https://www.ncbi.nlm.nih.gov/nuccore/MZ169558, Associated BioProject, https://www.ncbi.nlm.nih.gov/bioproject/730083, BioSample accession number at https://www.ncbi.nlm.nih.gov/biosample/SAMN19217413 and Sequence Read Archive at https://www.ncbi.nlm.nih.gov/sra/SRR14574595.
